# Comparative genomic analysis of the novel strain *Vibrio cidicii* VC01, isolated from China

**DOI:** 10.1128/spectrum.02209-25

**Published:** 2025-11-11

**Authors:** Fei Wu, Shifang Wang, Xue Li, Miaomiao Zhang, Xi Luo, Shenjie Ji, Renfei Lu

**Affiliations:** 1 Department of Clinical Laboratory, Affiliated Nantong Hospital 3 of Nantong University 66479, Nantong, Jiangsu, China; 2 Key Laboratory of Medicine, Nantong Center for Disease Control and Prevention 680464 , Nantong, Jiangsu, China; 3 Department of Clinical Laboratory, Qidong People’s Hospital, Qidong, Jiangsu, China; Gujarat Biotechnology University, Gandhinagar, Gujarat, India

**Keywords:** *Vibrio cidicii*, comparative genomics, O-antigen, Tn*7*-like transposon

## Abstract

*Vibrio cidicii* is an opportunistic pathogen that is widely distributed in the environment worldwide. Using whole-genome sequencing (WGS), we investigated the genetic characteristics of *V. cidicii* isolated from the seawater (named as *V. cidicii* VC01) by the Chinese Center for Disease Control and Prevention in Nantong, China. VC01 comprised two circular chromosomes, containing 4,013 potential coding sequences and 151 RNA genes. A phylogenetic tree constructed from the 16S rRNA genes of *Vibrio* spp. indicated that *V. cidicii* strains were closely related to *V. navarrensis* strains. The phylogenetic tree of single nucleotide polymorphisms revealed that 19 *V*. *cidicii* strains, including 18 *V*. *cidicii* strains from the National Center for Biotechnology Information database and VC01, were divided into three genotypes with VC01 belonging to genotype 2. Based on the genetic screening of virulence factors, the 19 strains were classified into nine functional categories, and the differences between strains were mainly in adherence and immune modulation. All *V. cidicii* strains carried six antimicrobial resistance genes (AMRs), while VC01 carried two additional AMRs (*dfrA3* and *qnrVC6*). We defined five *V. cidicii* O-genotypes (VCOg, named VCOg1–VCOg5) based on analyses of the O-antigen biosynthesis gene clusters (O-AGCs). The O-AGCs genotype of *V. cidicii* VC01 was VCOg4. Based on WGS, we found that VC01 carried a Tn*7*-like transposon with a type I restriction-modification system. Through comparative analysis, we first revealed the O-AGCs diversity in *V. cidicii* strains and the unique strain VC01 carrying a Tn*7*-like transposon with a type I restriction-modification system.

**IMPORTANCE:**

*Vibrio cidicii* strains are opportunistic pathogens causing bacteremia in humans and can be multidrug-resistant. Since few studies report on *Vibrio cidicii* strains, there is a lack of knowledge of the genetic diversity, presence of antimicrobial resistance genes, and virulence factors in *V. cidicii* strains. This study revealed the genomic features, phylogeny, and diversity of *V. cidicii* strains, highlighting five O-antigen biosynthesis gene cluster genotypes in *V. cidicii* strains and a Tn7-like transposon carrying a type Ⅰ restriction-modification in VC01. These findings provide new insights into the genetic diversity and antimicrobial potential of *V. cidicii* strains.

## INTRODUCTION

*Vibrio* is a gram-negative, facultative anaerobic bacterium widely distributed in the marine environment and generally requires sodium for growth (1). *Vibrio cidicii* is a rod-shaped and motile bacterium that was first identified by Orata (2016) in USA (2). *V. cidicii* is widely distributed in river water and seawater samples worldwide (2, 3). *V. cidicii* is an opportunistic pathogen that causes bacteremia in humans and can be multidrug-resistant (2).

The O-specific polysaccharides (O-antigens) covering the outermost layer of gram-negative bacteria are responsible for serological diversity. This variable constituent of the cell is arguably responsible for the virulence of the bacteria. O-antigen analysis is a key element in the serotyping of *Vibrio*. The O-antigens have been well studied in *Vibrio* spp. like *V. cholerae*, *V. parahaemolyticus*, and *V. metschnikovii* (4–6). In *V. metschnikovii* and *V. cholerae*, the genes that generate the O-antigen are distributed between the epimerase gene, *gmhD*, and the cleavage gene, *rjg*, which are involved in the major stages of lipopolysaccharide-core generation (4, 6). The O-genotypes have not been identified in *V. cidicii* strains. Thus, a systematic and large-scale analysis of *V. cidicii* O-antigen biosynthesis gene clusters (O-AGCs) is needed to better understand the diversity of its O-genotypes.

Tn*7* is a distinctive bacterial transposon composed of five genes, *tnsABCDE* (7). The dissemination of Tn*7* and related elements, called Tn*7*-like elements that contain homologs of the Tn*7* transposition proteins, is present in highly diverged bacteria adapted to a remarkable number of different environments (8, 9). In *V. cholerae* strains, the Tn*7*-like transposon consists of four genes: *tnsABC* and *tniQ* (10). TniQ shows homology to TnsD, which targets a sequence-specific site, *attTn7*, for insertion (11). Tn*7*-like transposon has not yet been reported in *V. cidicii*, leaving a significant gap in our understanding of its host range and evolutionary dynamics. Thus, we analyzed *V. cidicii* strains isolated in our study and from the National Center for Biotechnology Information (NCBI) database to explore the diversity and distribution of Tn*7*-like transposon, providing insights into its potential role.

The objectives of this study were to investigate the genomic features, genetic diversity, and evolutionary relationships of *V. cidicii* strains in our study and from the NCBI database, with a focus on the distributions of virulence factors (VFs), antimicrobial resistance genes (AMRs), O-antigens, and Tn*7*-like transposon. By analyzing *V. cidicii* strains that have not been systematically analyzed before, this study provides novel insights into the O-AGCs diversity and mobilization potential of Tn*7*-like transposon in *V. cidicii* strains.

## RESULTS

### The genomic characterization of VC01

*V. cidicii* VC01 was isolated from the seawater by the Center for Disease Control and Prevention (CDC) in Nantong, China. VC01 was initially recognized as *Vibrio vulnificus* based on colony morphology, salt tolerance, and bioMérieux VITEK 2 compact instrument (bioMérieux, Marcy-l’Étoile, France), and sequenced with *V. vulnificus* VV2018. The genome completeness and contamination of VC01 were 100% and 0.4%, respectively. The average nucleotide identity (ANI) value between VC01 and *V. vulnificus* ATCC 27562 (reference genome, ASM222426v1) was 85.52%, while the ANI value between VC01 and other *V. cidicii* strains available from the NCBI genome database was 98.27% (range: 98.07%–98.36%). Thus, the strain was identified as *Vibrio cidicii* and named VC01.

**Fig 1:**
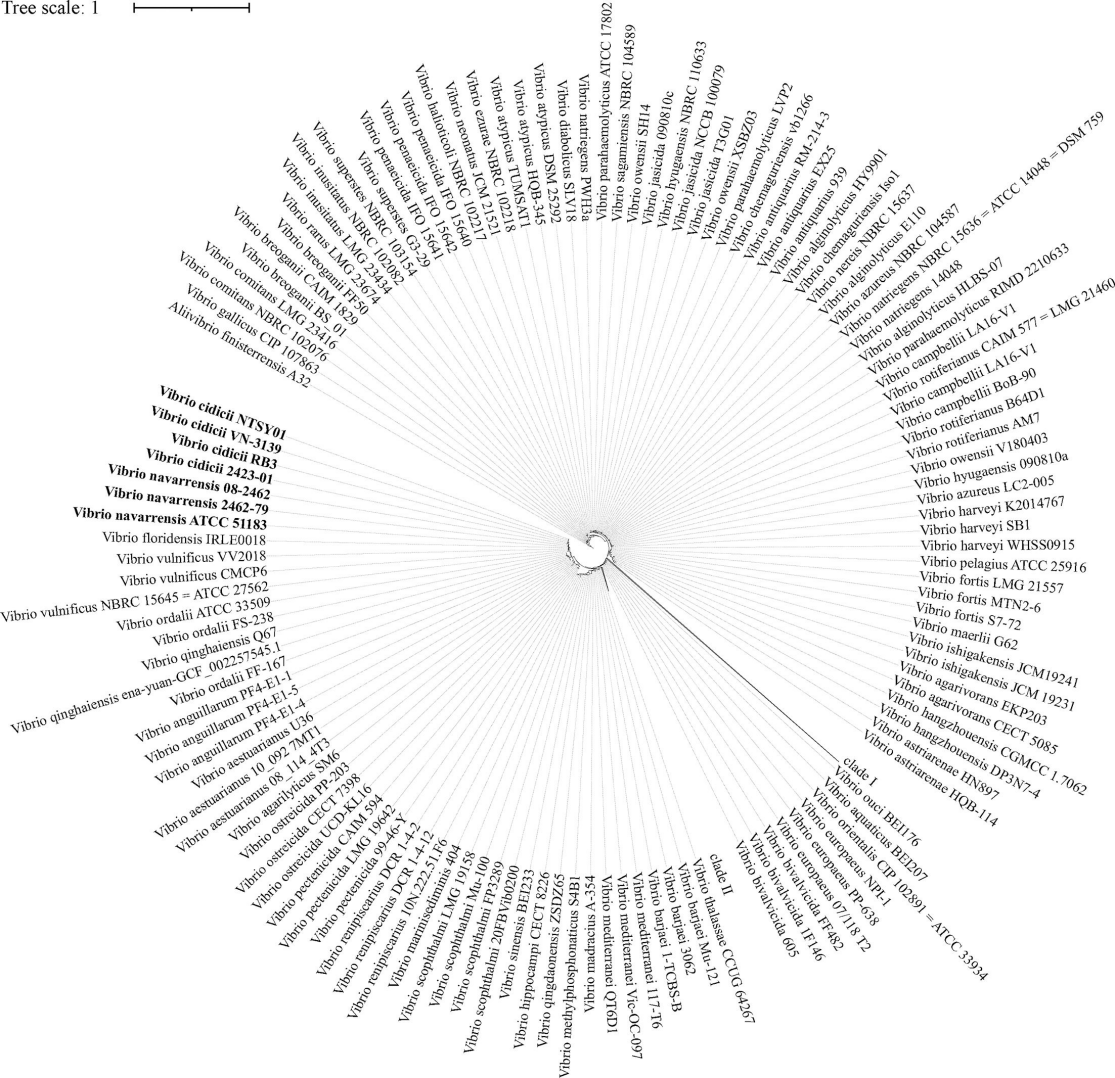
The 16S rRNA gene phylogenetic tree obtained in iTOL using the 16S rRNA gene sequences of *Vibrio* spp. and their related taxa using the maximum likelihood method. *Aliivibrio finisterrensis* A32 was used as the outgroup.

The complete genome of VC01 comprised two chromosomes, Chr I and Chr II. The Chr I consisted of 3,273,343 bp with a GC content of 48.08% containing 2,868 predicted coding sequences (CDSs), 105 tRNA genes, and 31 rRNA genes (Table 1T). The Chr II consisted of 1,363,011 bp with a GC content of 47.90% containing 1,145 predicted CDSs, 12 tRNA genes, and 3 rRNA genes (Table 1). A total of 330 representative sequences were used to investigate the phylogenetic relationships between *V. cidicii* and the other *Vibrio* spp. (Table S1). The phylogenetic tree of the 16S rRNA genes of representative *Vibrio* spp. indicated that *V. cidicii* strains were closely related to *V. navarrensis* strains (Fig. 1).

**TABLE 1:** Genomic characteristics of the *V. cidicii* VC01

Feature	Chr I	Chr II
Length (bp)	3,273,343	1,363,011
G+C content (%)	48.08	47.90
Predicted CDSs	2,868	1,145
Average length (bp)	965	1,031
Known proteins	1,825	601
Hypothetical proteins	1,043	544
Protein coding (%)	84.62	86.67
tRNA genes	105	12
16S rRNA genes	10	1
23S rRNA genes	10	1
5S rRNA genes	11	1

### Functional analyses of VC01

**Fig 2:**
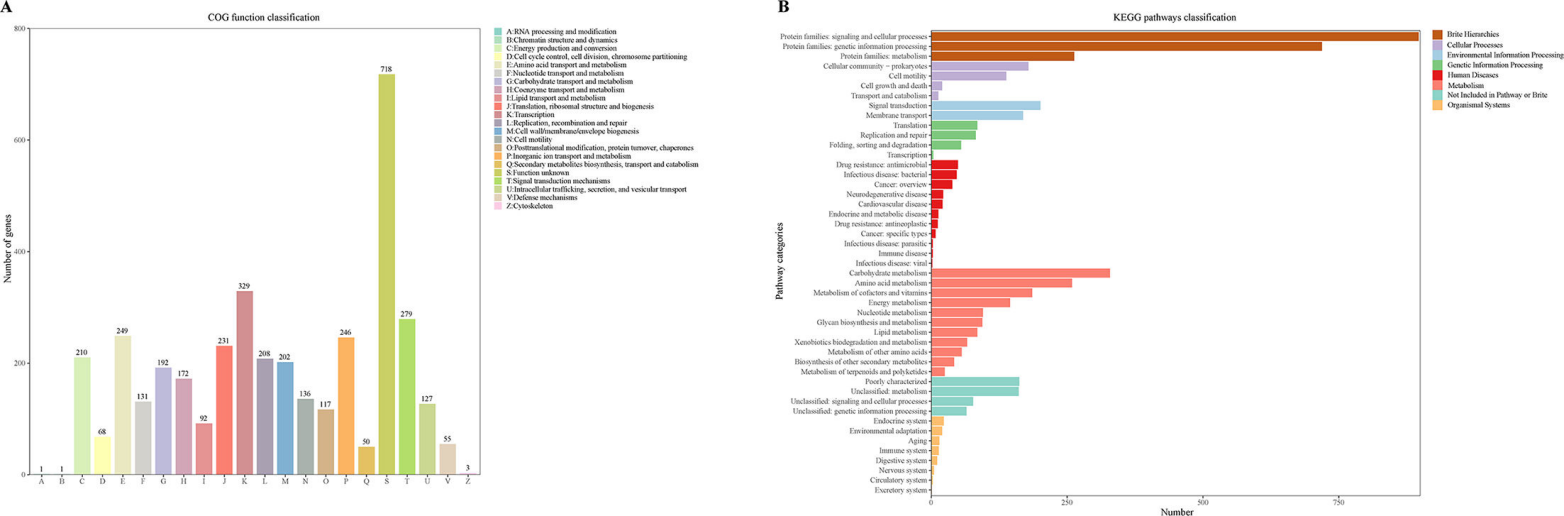
The functional annotation of CDSs in the whole genome of *V. cidicii* VC01. (**A**) The COG functional annotation of CDSs of *V. cidicii* VC01. (**B**) The KEGG functional annotation of CDSs of *V. cidicii* VC01.

The Cluster of Orthologous Groups (COG) and Kyoto Encyclopedia of Genes and Genomes (KEGG) annotations are shown in Fig. 2. The COG annotation of the VC01 genome involved 22 categories. The highest number of genes was identified with unknown functionality, suggesting the presence of potentially novel adaptive traits. The five other categories with the largest number of genes were transcription, signal transduction mechanisms, amino acid transport and metabolism, inorganic ion transport and metabolism, and translation, ribosomal structure, and biogenesis (Fig. 2A).

Based on KEGG annotations, the VC01 genome had eight major categories including 47 KEGG pathways. Among them, the BRITE hierarchies category contained the highest gene count (approximately 1,616), involving in signaling and cellular processes and genetic information processing, followed by the biosynthesis of metabolism (approximately 1,382), involving in carbohydrate metabolism and amino acid metabolism (Fig. 2B).

**Fig 3:**
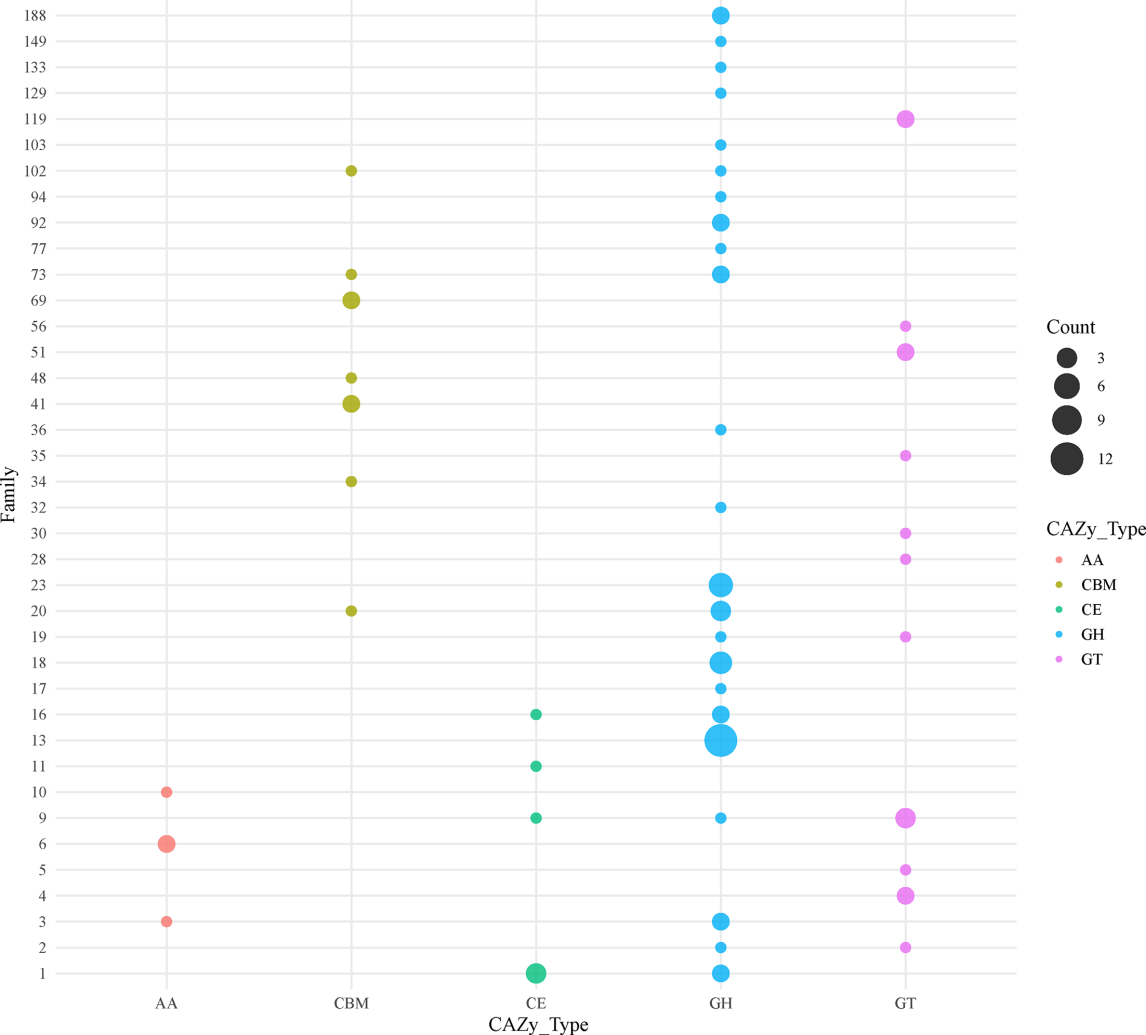
Family-wise distribution of different CAZyme classes in the genome of *V. cidicii* VC01. The different CAZyme classes shown are AA, auxiliary activities; CBM, carbohydrate-binding modules; CE, carbohydrate esterases; GH, glycoside hydrolases; and GT, glycosyltransferases.

The CAZyme repertoire of VC01 consisted of 78 unique proteins with 84 CAZyme domains (Fig. 3). The genome of VC01 was dominated by 49 glycoside hydrolases (GH), followed by 16 glycosyltransferases (GT), 9 carbohydrate-binding modules (CBM), 6 carbohydrate esterases (CE), and 4 auxiliary activity (AA) family proteins. The GHs, GTs, CBMs, and CEs belonged to 23, 11, 7, and 4 families, respectively. The AAs were classified into three families: families 3, 6, and 10. Among the GHs, GH13 was the dominant family, with 12 representative members involved in the hydrolysis of α-linkages in glucans.

### Comparative genomic analysis of VC01

**Fig 4:**
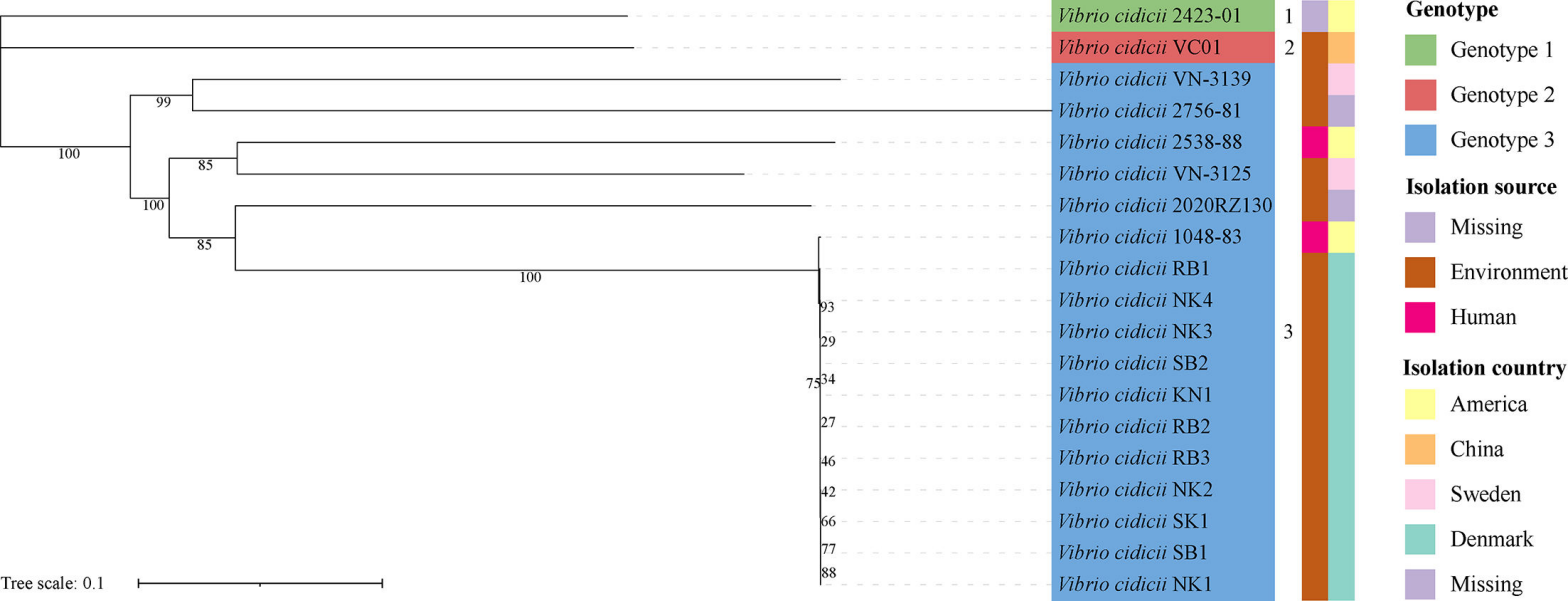
An unrooted maximum likelihood phylogenetic tree of *V. cidicii* VC01 with 18 other *V. cidicii* strains based on core genome SNPs.

A total of 19 *V*. *cidicii* strains, including VC01, were used to construct the phylogenetic tree based on the core single nucleotide polymorphisms (SNPs) (Table S2 and Fig. 4). Most strains (17/19, 89.47%) were isolated from the environment, while only two strains were isolated from humans in USA. The phylogenetic tree of core SNPs showed that 19 *V*. *cidicii* strains were divided into three genotypes, including genotype 1, genotype 2, and genotype 3. Genotype 1 and genotype 2 each contained only one strain: 2423-01 and VC01 strain, respectively. The other 17 *V*. *cidicii* strains were clustered in genotype 3, where the *V. cidicii* strains isolated from Denmark were all in a sub-lineage.

**Fig 5:**
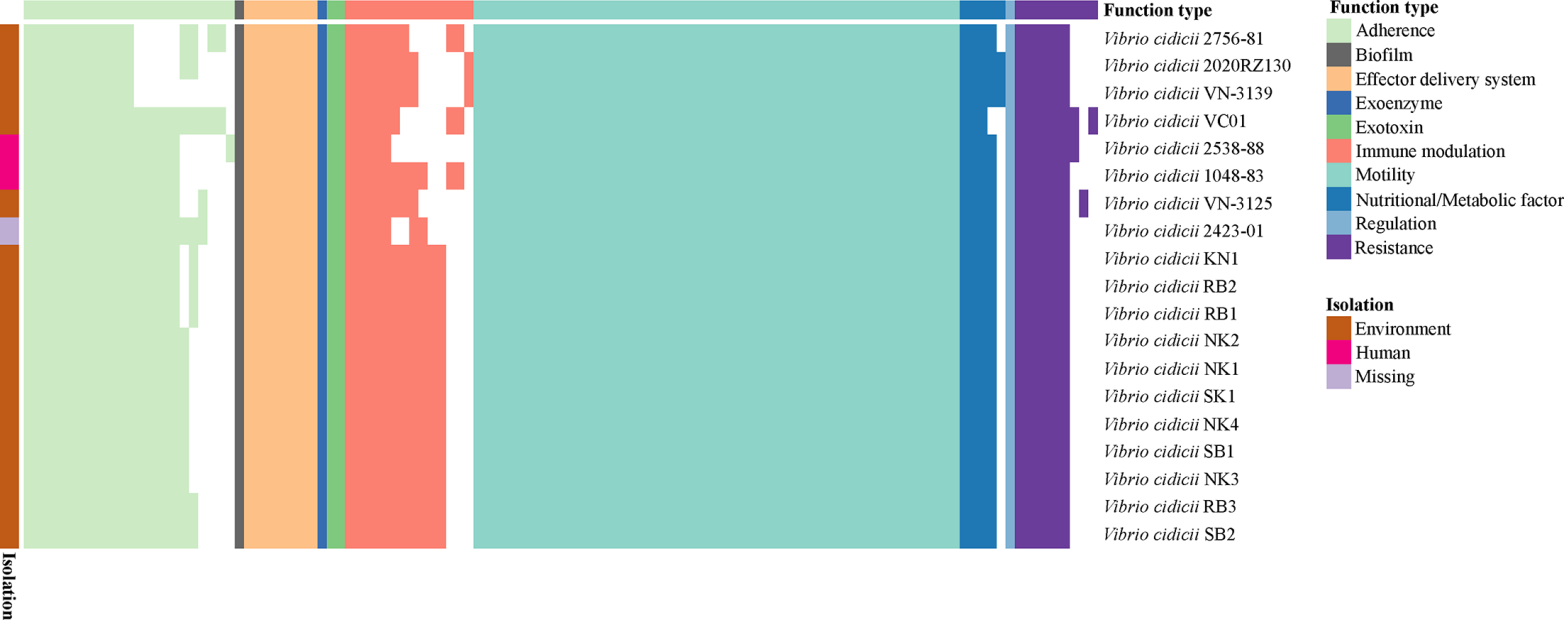
Presence/absence of VFs and resistance-related genes in each *V. cidicii* genome.

A total of 117 VFs were predicted among the 19 *V*. *cidici*i strains, and these VFs were mainly associated with motility (peritrichous flagella and polar flagella), adherence (MSHA pili and type IV pili), immune modulation (capsule and capsular polysaccharide), effector delivery system (Eps T2SS), and nutritional/metabolic factor (*viuPDGC* system) (Fig. 5). The largest difference among *V. cidicii* strains was in adherence and immune modulation, which likely affected their niche adaptation and survival in different environments. With the exception of the *mshA* gene, the VC01 strain encoded the most VFs associated with adherence compared to the other 18 *V*. *cidicii* strains. Compared to the other 18 *V*. *cidicii* strains, VC01 lacked VFs associated with immune modulation, including *rmlA*, *wbfUY*, *wecBC*, and *wcaJ*.

Nine AMRs were identified in the genome of 19 *V*. *cidicii* strains (Fig. 5). All 19 *V*. *cidicii* strains encoded six AMRs, including *tet*(35), *qnrVC1*, *msbA*, *rsmA*, *crp*, and *ugd*. The VC01 strain and 2538-88 strain encoded the *dfrA3* gene. The VC01 strain exclusively encodes the *qnrVC6* gene that is required to confirm the phenotypic impact of this gene, while the VN-3125 strain uniquely encodes the *aph6-Id* gene.

### O-AGCs of *V. cidicii*

**Fig 6:**
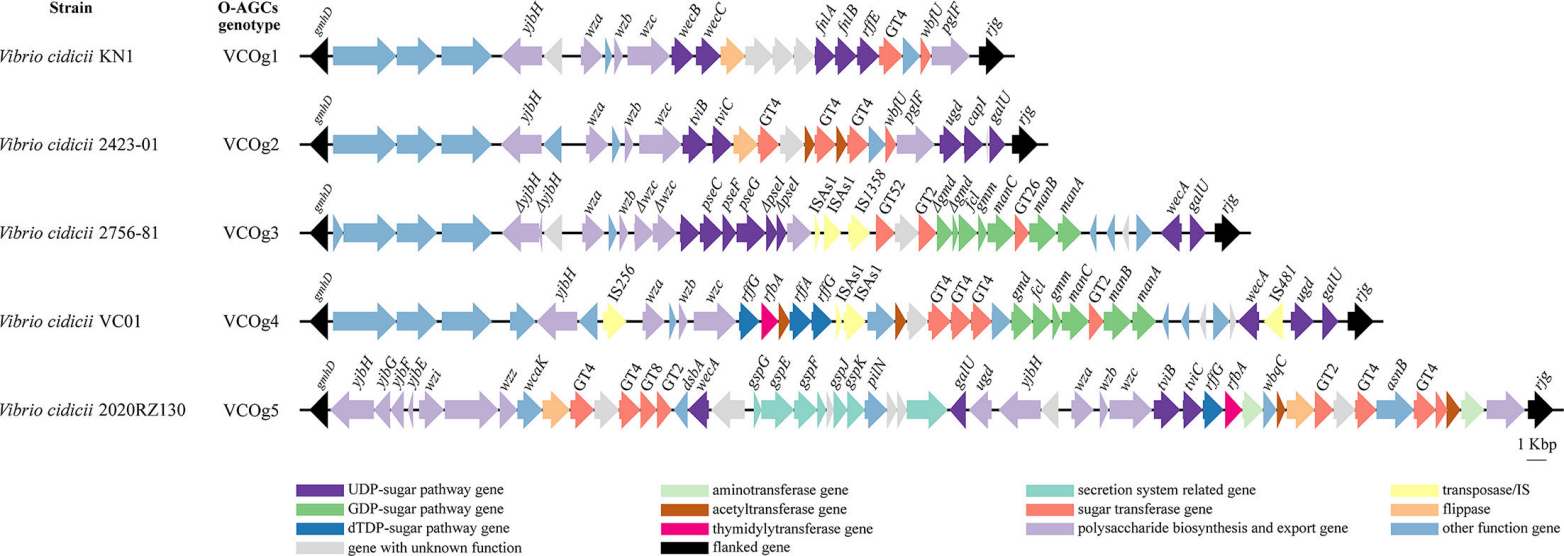
The genetic structures of the fiv*e V. cidicii* O-genotypes identified from the *V. cidicii* reference strains. The different colored arrows indicate the function, location, and direction of gene transcripts in the O-AGCs.

The 19 *V*. *cidicii* genome sequences were used to analyze the O-AGCs of *V. cidicii* strains (Table S2). Due to the draft genomes of some *V. cidicii* strains, only five *V. cidicii* genomes had contiguous, fully assembled sequences covering the region between *gmhD* and *rjg* (encoding epimerase and metallo-hydrolase). The five O-AGCs of *V. cidicii* strains were all different and grouped into five clearly distinguishable O-antigen genotypes based on their genetic organization (Fig. 6) and termed as VCOg1–VCOg5, respectively. The O-AGCs genotype of VC01 was VCOg4.

The O-AGCs in *V. cidicii* strains exhibited high diversity due to variations in their arrangement and organization (Table S3). However, they all contained three major classes of genes, namely nucleotide sugar biosynthesis genes, GT genes, and polysaccharide biosynthesis and export genes. Nucleotide sugar biosynthesis genes showed distinct distributions across clusters (Table S3). For putative glycosyltransferase genes, each genotype contained 1–6 genes, with genes in families 2, 4, and 8 being widely distributed. For polysaccharide biosynthesis and export genes, three conserved genes (*wza*, *wzb*, and *wzc*) as shown in Fig. 6, which are responsible for polymerization control and the translocation of CPS, were present in all genotypes (VCOg1–VCOg5).

The genes between *gmhD* and *rjg* were similar among VCOg1–VCOg4, with other functions (Table S3). Furthermore, the O-AGCs genotype of VCOg4 of the VC01 strain was very similar to VCOg3 of the 2756-81 strain. Besides the three major classes of genes, both VCOg3 and VCOg4 contained GDP-sugar pathway genes (*gmd*, *fcl*, *gmm*, *manA*, *manB*, and *manC*) responsible for the formation of GDP-D-Man, and some inserted sequences (IS, like IS*As1*).

### Comparative analysis of the Tn*7*-like transposon of VC01

**Fig 7:**
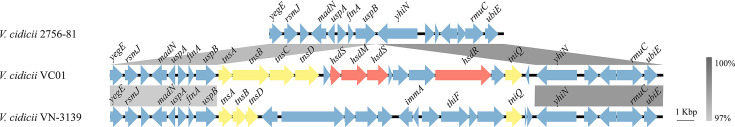
Comparative genomic analysis of the genetic context of Tn*7*-like genes in *V. cidicii* VC01 with the sequences of two other *V. cidicii* strains. The location and polarity of CDSs are shown with arrows. The extent of homologous regions is indicated by dark gray shading.

Based on comparative analysis with the 18 other *V. cidicii* strains, the Tn*7*-like transposon was found in the VC01 genome (Fig. 7). The sequence of the 2756-81 strain was used as the representative sequence for the sequences of the other 17 *V*. *cidicii* strains. The Tn*7*-like transposon in the VC01 strain was integrated downstream of *uspB* and upstream of *yhiN* (encoding NAD(P)/FAD-dependent oxidoreductase and bifunctional diguanylate cyclase/phosphodiesterase). The Tn*7*-like transposon in the VC01 and VN-3139 strains comprises the transposase genes, *tnsABCD*, and a target-site selector tniQ/tnsD. The transposon contained type I restriction-modification system subunits (*hsdS-hsdM-hsdS* and *hsdR*), phospholipase, nucleotidyltransferase, and other genes of unknown function in VC01. The Tn*7*-like transposon lacking tnsC in VN-3139 contained genes encoding ATP-binding protein, nucleotidyltransferase, thiamine, and Mov34/MPN/PAD-1 family protein. The sequences of *tnsABD* and *tniQ* showed high divergence in VC01 and VN-3139.

The 23 sequences of *Vibrio* genus with high identity compared with the Tn*7*-like transposons in NCBI database were used for further analysis (Table S4). Of 23 strains, 73.91% were *Vibrio parahaemolyticus*, all isolated from China, followed by *Vibrio cholerae* (13.04%), *Vibrio metoecus* (8.70%), and *Vibrio fluvialis* (4.35%). The 24 sequences, including VC01, were grouped into seven clusters. Most of the sequences (14/24, 58.33%) were grouped into cluster 2.

Seven sequences were used as representative sequences for further analyses (Fig. S1). All Tn*7*-like transposons contained *tnsABCD*, *hsdS-hsdM-hsdS*, *hsdR*, and *tniQ* except *Vibrio parahaemolyticus* C3_5 and *Vibrio parahaemolyticus* Vb2627. Despite low sequence identity, the downstream gene *hsdS* of *hsdM* was annotated with the same name as in other sequences, retaining the same functional designation. As shown in Fig. S1, the sequence of the Tn*7*-like transposon of VC01 was similar to that of *Vibrio fluvialis* VF035 isolated from a fish in Shandong, China. Because the genome of *V. fluvialis* VF035 was a draft, the *tnsA* gene was truncated, and the genes upstream of *tnsA* were not found. The gene downstream of the Tn*7*-like transposon in VC01 was *yhiN*, while there were *araH* and *araG* in *V. fluvialis* VF035.

### Comparative analysis of Tn*7*-like sequences

**Fig 8:**
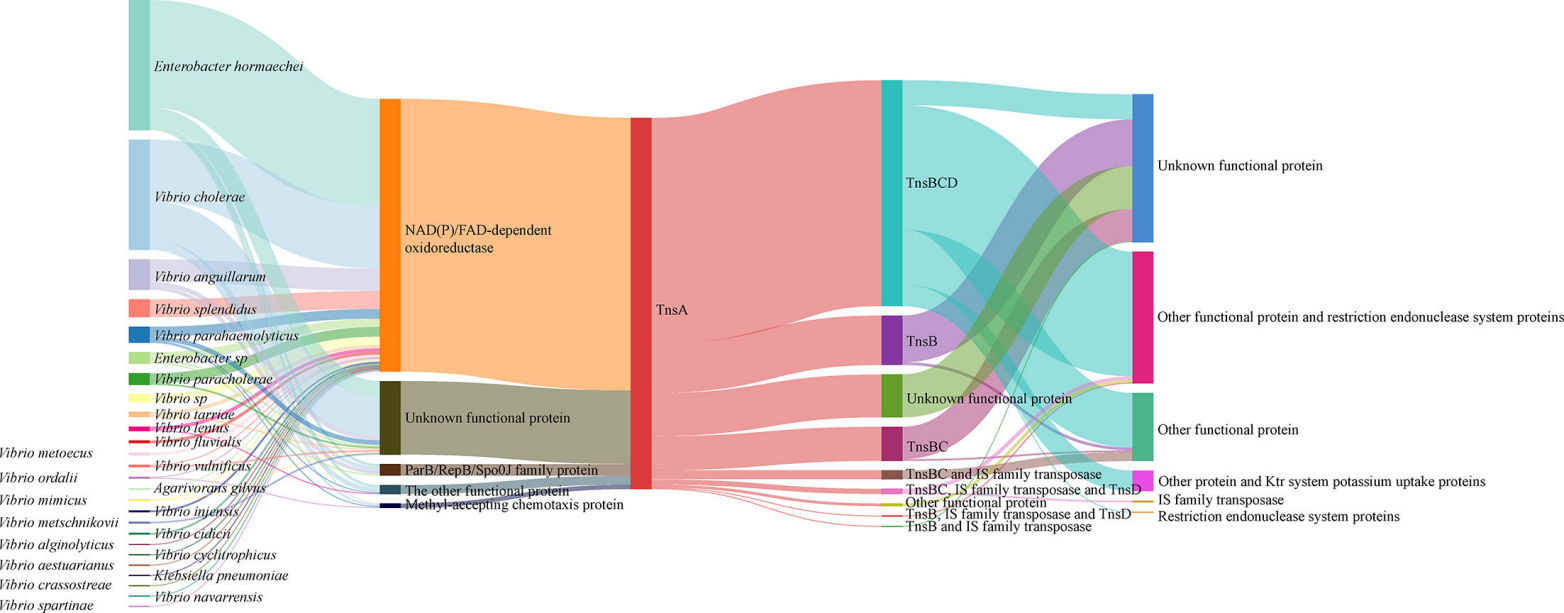
Sankey diagrams for the function of genes upstream and downstream of TnsA among different strains. The length of the columns represents the proportion of the isolates. The thicker the line, the greater the number of isolates involved.

A total of 413 sequences containing *tnsA* were downloaded from the NCBI database for further analysis (Table S5). Only one *V. cidicii* strain, *V. cidicii* VN-3139, encoding *tnsA* was found in the database. Most of the strains encoding *tnsA* were *Enterobacter hormaechei* (145, 35.02%) and *Vibrio cholerae* (123, 29.71%). The *tnsA* was positioned in the genomic region flanked by the genes encoding NAD(P)/FAD-dependent oxidoreductase and TnsBCD in most sequences (93, 22.46%) (Fig. 8). All genes encoding restriction endonuclease system proteins were downstream of *tnsABCD*. Aside from restriction endonuclease system genes, the genes (*ktrA* and *ktrB*) encoding the Ktr system potassium uptake proteins were found downstream of *tnsABCD* in some strains, such as *Vibrio cholerae* and *Vibrio anguillarum*.

## DISCUSSION

Genomic analysis confirmed that VC01 belongs to *V. cidicii*, consistent with established species delineation criteria (12). Until now, only two studies have reported the isolation and genomic features of *V. cidicii* strains (2, 3). This is the first report of a comparative genomic analysis of VC01 strain isolated from Nantong, China. The genome of the VC01 strain consisted of two chromosomes without plasmids, as determined by PlasmidFinder. The phylogenetic tree of 16S rRNA genes showed that the *V. cidicii* strains were closely related to *V. navarrensis* strains, corresponding with the result of the phylogenetic tree of four housekeeping genes and whole-genome variations in *Vibrio* spp. (2). As few reports studied the function of *V. cidicii* genes, about 40% of genes in VC01 had unknown functions. Thus, further investigation is needed to elucidate the functions of the remaining genes of unknown function, using approaches such as gene knockouts or overexpression, transcriptomic and proteomic analyses, and protein functional assays.

The COG and KEGG analysis of the CDSs of VC01 indicated that most of the genes were assigned to the functional categories for transcription, cellular signaling, and metabolism (especially carbohydrate metabolism and amino acid metabolism), which are frequently associated with bacterial life cycle activities and energy metabolism; thus, suggesting that they possess a strong ability to metabolize amino acids and carbohydrates. Those findings further corroborated the importance of carbohydrates as an energy source for the biological functions of VC01 strains (13). The GH family of genes plays an important role in a variety of biological processes including sugar biosynthesis (14). The GH13 was the dominant GH family in the VC01 strain, indicating their potential to degrade starch and pullulan (15). The VC01 strain encoded the most VFs associated with adherence (including *mshCD*) and did not encode the *mshA* gene. A previous study reported that *Vibrio parahaemolyticus* strains with *mshCD*, but lacking *mshA*, were identified as strong biofilm-forming strains (16). Compared with 18 other *V. cidicii* strains, the *gmd* gene was found in three *V. cidicii* strains including VC01. GMD catalyzes the conversion of GDP-D-mannose to GDP-4-dehydro-6-deoxy-D-mannose (17). The *gmd* gene was also a part of O-AGCs (VCOg3 and VCOg4) that can modulate c-di-GMP levels (18). The absence of VFs associated with the immune modulation of VC01 (*rmlA*, *wbfUY*, *wecBC*, and *wcaJ*) may exhibit potential toxic effects via adherence or antiphagocytosis or act directly as toxins (19). Six antibiotic resistance genes (*crp*, *ugd*, *msbA*, *rsmA*, *tet*(35), and *qnrVC1*) were found in all *V. cidicii* genomes. Those genes might increase the resistance of *V. cidicii* strains to macrolides, penams, fluoroquinolones, polymyxins, colistins, nitroimidazoles, diaminopyrimidines, phenicols, tetracyclines, and quinolones (20, 21). The VC01 strain encoded a quinolone resistance gene (*qnrVC6*) and a trimethoprim resistance gene (*dfrA3*) (22, 23). The ISNCY family transposase was detected downstream of *qnrVC6*, suggesting that ISNCY and *qnrVC6* might be transmitted via horizontal gene transfer. The O-AGCs based on *Vibrio* serotyping have become the “gold standard” for the clinical detection and epidemiological surveillance of human *Vibrio* pathogens (24). We defined five O-genotypes, named VCOg1–VCOg5, respectively. Assembly errors from short reads or variations resulting from different assembly methods require attention (25, 26). A potential mechanism for recombination and deletion events could also contribute to the antigenic diversity (27). We found that *V. cidicii* had considerable O-antigen diversity, suggesting that the genetic differences among those strains were also representative. Conversely, 14 *V*. *cidicii* strains had O-genotypes still to be identified in future work as their genome sequences were drafts. Although our findings suggested that the natural O-AGCs diversity among *V. cidicii* strains may be broader than currently recognized, this hypothesis required further validation. Future studies employing whole-genome sequencing and systematic screening of *V. cidicii* isolates will be essential to comprehensively assess the extent of O-AGCs diversity.

The Tn*7* and Tn*7*-like transposons are widely dispersed in bacteria, such as *Acinetobacter baumannii*, *Burkholderia*, *Pseudomonas syringae*, and *Vibrio cholerae* (28–31). The cargo genes, including antibiotic resistance gene cassettes, heavy metal resistance genes, iron-sequestering siderophores, nonribosomal peptide synthases, restriction-modification enzymes, and many other genes of unknown function, were transferred by Tn*7* and Tn*7*-like transposons (8, 9, 30, 32). By comparative analyses, we found that Tn*7*-like transposons were widely dispersed in *Vibrio* spp. and other strains, such as *Enterobacter hormaechei* and *Klebsiella pneumoniae*. The cargo genes encoding the proteins of unknown function, restriction endonuclease systems, and Ktr system potassium uptake proteins were carried by Tn*7*-like transposons. In *Vibrio cholerae*, the type I restriction-modification system is also associated with Tn*7*-like transposons (31). The acquisition of a restriction-modification system is indicative of traits that may be critical for bacterial fitness, either when competing in a polymicrobial setting (e.g., the gut) or in its native aquatic environment (31).

### Conclusion

This study investigated the genomic features of VC01 isolated from seawater in Nantong, China. It is the first study of the function of genes in VC01 and the diversity of VFs and AMRs among *V. cidicii* strains using comparative analysis. The phylogenetic tree of the core SNPs showed that 19 *V*. *cidicii* strains were divided into three genotypes. The VC01 strain belonged to genotype 2. We first reported and revealed the genomic structure of five O-AGCs of *V. cidicii* strains (VCOg1–VCOg5). We first found that the Tn*7*-like transposon carried a type I restriction-modification system in VC01. In addition, we found that Tn*7*-like transposons carried different cargo genes among bacteria.

## MATERIALS AND METHODS

### Bacterial strain and genomic DNA extraction

VC01 was isolated from the seawater by the CDC in Nantong, China, in 2019. The VC01 strain was initially recognized as *Vibrio vulnificus*, and was identified by ANI analysis. The genomic DNA of VC01 was extracted using a TIANamp Bacteria DNA Kit (Tiangen Biotech Company Ltd., Beijing, China) according to the manufacturer’s protocol.

### Genomic DNA sequencing, assembly, and annotation

The extracted genomic DNA of VC01 was sequenced using the PacBio Sequel system (Pacific Biosciences, Menlo Park, CA, USA) and Illumina HiSeq X Ten system (Illumina, San Diego, CA, USA). The PacBio long reads were assembled using Hifiasm v0.13-r308 and Canu v2.2 to ensure a robust genome assembly from long-read data (33, 34), and the Illumina short reads were mapped onto the assembled contigs to correct the primary assembly and control assembly quality using Pilon 1.22 and Quiver (35, 36). The genomic completeness and contamination of VC01 were evaluated by CheckM2 v1.0.1 with default settings (37). The rRNA and tRNA sequences were annotated using RNAmmer and tRNAscan-SE, respectively (38, 39). The potential CDSs were predicted and annotated by Prokka v1.14.6 due to its efficiency and accuracy in bacterial genome annotation (40). The CDSs of interest were annotated manually using the UniProtKB/SWISS-Prot database (41). KEGG and COG were analyzed using KofamScan v1.3.0 and eggNOG-mapper (http://eggnog-mapper.embl.de/), respectively (42, 43). The putative VFs and AMRs were identified using the virulence factors database (VFDB_setB) by BLASTx and the Resistance Gene Identifier v6.0.3 (https://github.com/arpcard/rgi) against the Comprehensive Antibiotic Resistance Database, respectively, because they are widely recognized and regularly updated resources for AMRs and VFs (44, 45).

### Phylogenetic analysis

A total of 330 representative sequences of *Vibrio* and *Aliivibrio finisterrensis* A32 used as outgroup were downloaded from the NCBI database to construct the phylogenetic tree of 16S rRNA genes. The sequences of the 16S rRNA genes were annotated using RNAmmer. The 16S rRNA genes were aligned using MAFFT v7.520 and trimmed using trimAl v1.4 (46, 47). The maximum likelihood (ML) phylogenetic analysis of the 16S rRNA genes was performed using IQ-TREE v2.2.6 with the TPM3+I+R10 model (1,000 bootstraps) and illustrated by iTOL v6.6 (48, 49).

### Comparative genomic analysis

A total of 18 available *V. cidicii* genome sequences, 24 *Vibrio* spp. sequences, and 413 sequences encoding *tnsA* were downloaded for further analysis. The whole-genome ANI between pairwise *V. cidicii* strains was calculated by the average_nucleotide_identity.py program in the pyani packages (https://github.com/widdowquinn/pyani). The core genome of these strains was produced by Harvest v1.1.2 using the *Vibrio cidicii* 2423-01 genome as the reference (50). Recombination events were removed from the core-genome alignment using Gubbins v2.2.0 (51). SNPs were then extracted from the recombination-free core genome alignment using SNP-sites v2.4.0 (52). The ML phylogenetic tree of SNPs was constructed using RAxML v8.2.12 in the GTRGAMMA model (1,000 bootstrap) and illustrated by iTOL v6.6 (53). The *gmhD* and *rjg* were used as marker genes to analyze the O-AGCs of *V. cidicii* strains. The CD-HIT was used to cluster the retained sequences with identity of 90% and coverage of 90% (54). Comparisons of the O-AGC sequences and Tn*7*-like region sequences were visualized using EasyFig v2.2.5 (55).

## Data Availability

The genome sequences of *V. cidicii* VC01 are deposited in the NCBI database under BioProject accession number PRJNA1284038.

## SUPPLEMENTAL MATERIAL

Fig. S1 spectrum.02209-25-s0001.pdf Comparative analysis of the Tn7-ike regions of seven representatives from 24 sequences.

Table S1 spectrum.02209-25-s0002.xlsx The 16S rRNA gene of representative strains downloaded from the NCBI database.

Table S2 spectrum.02209-25-s0003.xlsx Information concerning *V. cidicii* strains from the NCBI database.

Table S3 spectrum.02209-25-s0004.xlsx The gene clusters and their key genes with predicted function in the O-AGCs genotype.

Table S4 spectrum.02209-25-s0005.xlsx The details of *Vibrio* spp. containing Tn7-like sequences.

Table S5 spectrum.02209-25-s0006.xlsx The sequences containing *tnsA* downloaded from the NCBI database.

ASM does not own the copyrights to Supplemental Material that may be linked to, or accessed through, an article. The authors have granted ASM a non-exclusive, world-wide license to publish the Supplemental Material files. Please contact the corresponding author directly for reuse.
