# Supplementary material for: Comparative genomic analysis of the novel strain Vibrio cidicii VC01, isolated from China
Source: Microbiol Spectr. 2025 Nov 11;13(12):e02209-25. doi: 10.1128/spectrum.02209-25 (PMC12671221; doi:10.1128/spectrum.02209-25)
Supplement: Fig. S1 — Comparative analysis of the Tn7-ike regions of seven representatives from 24 sequences. [file spectrum.02209-25-s0001.pdf]

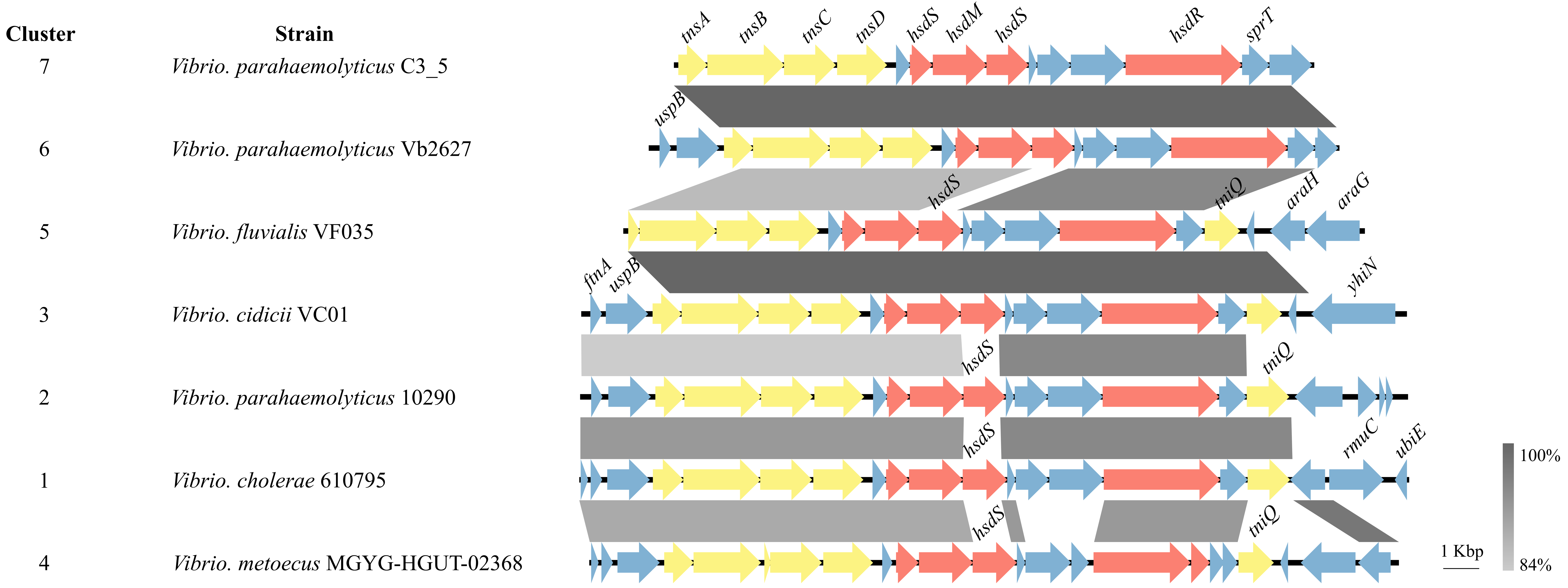

Figure S1. Comparative analysis of the Tn7-ike regions of seven representatives from 24 sequences. The direction of genes is indicated by an arrow. Homologous genes are shown in the same color.
